# Biogeographic Variation in Host Range Phenotypes and Taxonomic Composition of Marine Cyanophage Isolates

**DOI:** 10.3389/fmicb.2016.00983

**Published:** 2016-06-24

**Authors:** China A. Hanson, Marcia F. Marston, Jennifer B. H. Martiny

**Affiliations:** ^1^School of Biological and Chemical Sciences, Queen Mary University of London, LondonUK; ^2^Department of Ecology and Evolutionary Biology, University of California, Irvine, Irvine, CAUSA; ^3^Department of Biology and Marine Biology, Roger Williams University, Bristol, RIUSA

**Keywords:** cyanophage, biogeography, host range, marine, *Synechococcus*, T4-like phage, myovirus, podovirus

## Abstract

Despite the important role of phages in marine systems, little is understood about how their diversity is distributed in space. Biogeographic patterns of marine phages may be difficult to detect due to their vast genetic diversity, which may not be accurately represented by conserved marker genes. To investigate the spatial biogeographic structure of marine phages, we isolated over 400 cyanophages on *Synechococcus* host strain WH7803 at three coastal locations in the United States (Rhode Island, Washington, and southern California). Approximately 90% of the cyanophage isolates were myoviruses, while the other 10% were podoviruses. The diversity of isolates was further characterized in two ways: (i) taxonomically, using conserved marker genes and (ii) phenotypically, by testing isolates for their ability to infect a suite of hosts, or their “host range.” Because host range is a highly variable trait even among closely related isolates, we hypothesized that host range phenotypes of cyanophage isolates would vary more strongly among locations than would taxonomic composition. Instead, we found evidence for strong biogeographic variation both in taxonomic composition and host range phenotypes, with little taxonomic overlap among the three coastal regions. For both taxonomic composition and host range phenotypes, cyanophage communities from California and Rhode Island were the most dissimilar, while Washington communities exhibited similarity to each of the other two locations. These results suggest that selection imposed by spatial variation in host dynamics influence the biogeographic distribution of cyanophages.

## Introduction

Although biogeography has been a cornerstone of biology for over a century, it is only within the last several decades that microorganisms have been studied in this context (reviewed in Martiny et al., 2006). A recent review of this body of work shows that like larger organisms, nearly all free-living microorganisms exhibit biogeographic structure (Hanson et al., 2012). In contrast, much less is known about the distribution of viruses and particularly, those that infect bacteria (bacteriophages). Even basic spatial patterns of bacteriophages in natural environments remain unresolved (Thurber, 2009; Díaz-Muñoz and Koskella, 2014).

Based on initial surveys, marine bacteriophages were thought to be widely distributed and lacking in spatial distribution patterns (Breitbart and Rohwer, 2005). Both isolation-based and direct sequencing studies have found many “ubiquitous” phage taxa that are present in samples from distant locations and diverse environments (Kellogg et al., 1995; Zhong et al., 2002; Breitbart et al., 2004; Short and Suttle, 2005; Angly et al., 2006; Goldsmith et al., 2011), or have reported phage distributions that appear unrelated to geographic distance and environmental variation (Huang et al., 2010; Jameson et al., 2011). At small spatial scales such as within a water body or coastal region, studies have also found that the composition of phages infectious to cyanobacteria (cyanophages) varies little (Wang and Chen, 2004; Clasen et al., 2013).

Evidence is gradually emerging that at least some marine phages are unevenly distributed in space, challenging the notion that they lack biogeographic patterns (Wichels et al., 2002; Thurber, 2009; Marston et al., 2013; Huang et al., 2015). For example, the distribution of single-stranded DNA phages in the North Atlantic is related to geographic distance; communities closer together tend to be more similar than those far apart (Tucker et al., 2011). Additionally, marine cyanophage composition differs at both local and regional scales (Marston et al., 2013) and is related to salinity (Sullivan et al., 2008) and depth (Frederickson et al., 2003).

Two methodological factors may contribute to the lack of identified biogeographic patterns for phages. First, given the recent evidence for strong temporal patterns in marine microbial diversity (Fuhrman et al., 2006; Gilbert et al., 2009, 2012; Giovannoni and Vergin, 2012), including phages (Marston and Sallee, 2003; Mühling et al., 2005; Chen et al., 2009; Chow and Fuhrman, 2012; Clasen et al., 2013; Marston et al., 2013; Pagarete et al., 2013), variation over space may be obscured by temporal variation when multiple locations are sampled at different points in time. Second, the ability to detect spatial patterns depends on both the taxonomic resolution examined and the genetic region or phenotypic trait used to characterize diversity (Hanson et al., 2012). It is well-recognized, for instance, that bacteria defined as the same operational taxonomic unit (OTU) based on 16S nucleotide sequence similarity may have divergent genotypes at other loci or may differ in gene content, leading to differences in phenotype and, therefore, niche preference (Stackebrandt and Goebel, 1994; Moore et al., 1998; Jaspers and Overmann, 2004; Gevers et al., 2005; Hahn and Pockl, 2005). Thus, studies using highly conserved marker genes to characterize microbial diversity may miss ecologically relevant patterns.

Diversity at the sub-OTU level, often termed “microdiversity” (Moore et al., 1998; Acinas et al., 2004; Thompson et al., 2005; Buttigieg and Ramette, 2015), is gaining recognition as an important aspect of the ecology and evolution of phages (Marston and Amrich, 2009; Rodriguez-Brito et al., 2010; Deng et al., 2014). One trait that could be representative of phage microdiversity is host range, the breadth and identity of host strains on which a phage is capable of replicating. In marine cyanophages, which are a good study system for host–phage dynamics (Stoddard et al., 2007; Marston et al., 2012), host range can be altered by small changes, even point mutations, in genes involved in host recognition and attachment (Tetart et al., 1998; Scanlan et al., 2011). However, the genes responsible for host range remain cryptic for most marine phages (Angly et al., 2009; Breitbart, 2012; Marston et al., 2012; Martiny et al., 2014). Since phages are obligate parasites, there is presumably strong selection to overcome host resistance, and this selection likely acts independently of selection to maintain function in conserved marker genes (Rodriguez-Brito et al., 2010; Avrani et al., 2012). Thus, host range phenotypes may be highly variable among isolates that are indistinguishable based on marker gene sequence. This is supported by findings that host range phenotypes are not well correlated with phylogeny or taxonomic identity in cyanophages (Stoddard et al., 2007; Sullivan et al., 2008). Moreover, host range tends to be highly variable among marine isolates (Suttle and Chan, 1993; Waterbury and Valois, 1993; Lu et al., 2001; Holmfeldt et al., 2007; Dekel-Bird et al., 2014) and has been shown to vary non-randomly in space or time (Moebus and Nattkemper, 1981; Wichels et al., 2002; Comeau et al., 2005, 2006; Flores et al., 2013; Castillo et al., 2014). Only a few studies have investigated the biogeography of marine bacteria using both susceptibility to phages and taxonomic diversity of the bacteria (Holmfeldt et al., 2007; Castillo et al., 2014). We know of just a single study that has systematically done the same from the perspective of the phage (Comeau et al., 2006); i.e., compared spatial patterns in host range with taxonomic identity in marine phage isolates.

To investigate the spatial biogeography of marine phages, we isolated cyanophages infectious to the marine cyanobacterium, *Synechococcus* sp. WH7803, from three coastal locations in the U.S. within approximately 1-month time (August to September 2010). Sampling locations incorporated both the Atlantic (Rhode Island, henceforth referred to as “RI”) and the Pacific (southern California, “CA”; and Washington, “WA”) coasts. Cyanophage composition from each location was compared by characterizing the isolates both taxonomically using the sequence of a conserved marker gene (the *g20* portal protein gene for *Myoviridae*; or the DNA polymerase gene for *Podoviridae*), as well as phenotypically based on their ability to infect a suite of hosts (henceforth termed “host range”). These data were then analyzed to address the following question: are marine cyanophage communities spatially structured with respect to either their taxonomic composition or their host range phenotypes? Although it is not clear what genes are responsible for host recognition and attachment in cyanophages, there is no evidence to suggest that the *g20* or DNA polymerase genes are involved in host interactions (Tetart et al., 1998; Marston et al., 2012). Also, since selection to infect coexisting hosts likely operates independently of selection on the conserved marker genes, host range phenotypes may reveal ecological diversity that cannot be resolved by marker genes. Thus, we hypothesized that spatial variation in phage community composition would be more apparent by host range phenotype than by genetic taxonomy. In other words, we expected host range phenotypes to vary among locations, even if taxonomic composition did not.

## Materials and Methods

### Environmental Sampling

Surface seawater samples were collected from 11 sites in three different coastal regions of the U.S. between August 18, 2010 and September 20, 2010 (**Table 1**). The CA location consisted of five coastal sites [three sites in Orange County and two sites in the Tijuana River National Estuarine Research Reserve (NERR)], with a maximum distance of 136 km between sites. We have previously reported that although cyanophage OTU composition varies temporally on a seasonal scale at these sites within CA, cyanophage OTU composition does not vary by site within a season or month [based on g20 OTU composition of isolates; analysis of similarity (ANOSIM): *R* = 0.12, *p* = 0.13; Clasen et al., 2013). For the present study, each of the CA sites was sampled during two different time points in order to sufficiently capture the cyanophage diversity present during this 1-month sampling window. The WA location consisted of three sites in and near the Padilla Bay NERR (with a maximum distance apart of 15.4 km), while the RI location consisted of three sites in and near Narragansett Bay NERR (with a maximum distance apart of 26.5 km). Sites at these locations were sampled once during this period (WA: August 20 and RI: September 9). The approximate geodesic distance between locations, in order of furthest to nearest is: RI–CA 4160 km; RI–WA 4030 km; WA–CA 1800 km.

**Table 1:** Site characteristics and sampling dates.

Location	Sampling site	Coordinates	Date sampled	No. of cyanophage isolates obtained (no. of Myo; no. of Podo)^a^	WH7803 cyanophage abundance (10^2^ phage ml^-1^ ± SE)^b^	Cyanobacteria abundance (10^3^ cells ml^-1^ ± SE)
California (CA)	Crystal Cove State Park (CC)	33°34′24.7′′N, 117°50′23.7′′W	8/18/2010	34 (34; 0)	0.42 ± 0.18	34.6 ± 4.75
			9/15/2010	42 (42; 0)	2.83 ± 0.70	35.4 ± 3.84
	Newport Beach Pier (NB)	33°35′54.1′′N, 117°54′4.3′′W	8/18/2010	39 (39; 0)	0.16 ± 0.06	36.1 ± 6.07
			9/15/2010	39 (39; 0)	0.12 ± 0.003	25.7 ± 6.34
	Seal Beach (SB)	33°44′14.6′′N, 118°6′28.2′′W	8/18/2010	35 (35; 0)	0.13 ± 0.05	28.2 ± 2.42
			9/15/2010	34 (34; 0)	0.11 ± 0.03	16.0 ± 0.97
	Tijuana River, Boca Rio Channel (TA)	32°33′33.6′′N, 117°7′42.5′′W	9/2/2010	3 (3; 0)	0.41 ± 0.02	5.78 ± 0.24
			9/20/2010	8 (8;0)	7.67 ± 1.59	6.60 ± 1.67
	Tijuana River, Surf Zone (TB)	32°33′46.1′′N, 117°7′55.8′′W	9/2/2010	4 (4; 0)	0.06 ± 0.01	16.5 ± 0.35
			9/20/2010	32 (32; 0)	0.20 ± 0.03	5.34 ± 0.83
Washington (WA)	Washington Park (PA)	48°30′1.7′′N, 122°41′31.6′′W	8/20/2010	34 (25; 9)	0.86 ± 0.17	6.11 ± 0.64
	Samish Island (PB)	48°34′44.1′′N, 122°32′30.1′′W	8/20/2010	16 (13; 3)	1.95 ± 0.74	4.31 ± 1.33
	Padilla Bay (PC)	48°29′33.4′′N, 122°28′59.8′′W	8/20/2010	3 (3; 0)	4.37 ± 0.99	1.82 ± 0.33
Rhode Island (RI)	Colt State Park (CP)	41°41′6′′N, 71°17′44′′W	9/9/2010	36 (18; 18)	45.7 ± 4.18	79.5 ± 10.25
	Newport (NT)	41°27′2′′N, 71°21′5′′W	9/9/2010	8 (7; 1)	60.0 ± 10.0	38.2 ± 1.53
	Roger Williams University (RWU)	41°38′59′′N, 71°15′24′′W	9/9/2010	35 (22; 13)	33.3 ± 1.33	15.5 ± 1.47

*^a^Myo, myovirus; Podo, podovirus.*

*^b^Cyanophages infectious to *Synechococcus* WH7803, based on most probable number assays.*

For each sampling event, three replicate 1 l samples were collected between 7:00 and 13:00 at each site, transported in the dark, and processed for cyanophages within 12 h of collection. Temperature, pH, and salinity were measured on site with a handheld meter (Hanna Instruments; Supplementary Table S1). For nutrient analysis, duplicate subsamples (50 ml) were filtered through sterile 0.22 μm nylon syringe filters on site at the time of sampling into acid washed, sterile vials. Nutrient samples were transported in the dark on ice, and upon arrival to the lab were stored at -20°C. Nutrient estimates (PO_4_, NO_3_ + NO_2_, NO_2_, NH_4_, and SiO_4_) were performed using flow injection analysis by the Marine Science Institute Analytical Laboratory at University of California Santa Barbara (Supplementary Table S1).

### Cyanobacteria Enumeration

For all sites and dates, 30 ml of seawater was filtered onto a black 0.2-μm pore-sized polycarbonate filter. This was repeated for three replicate seawater samples for all sites, except RWU and all WA sites for which two replicate seawater samples were used. Each filter was filtered to dryness, mounted onto a glass slide with 100% glycerol and frozen (-20°C) until cells were enumerated by epifluorescence microscopy. Abundance (cyanobacterial cells ml^-1^) was determined by counting a total of >130 autofluorescing cells over at least 12 randomly chosen fields under wide-green excitation on a Zeiss Axioplan epifluorescence microscope (Carl Zeiss Microscopy, Oberkochen, Germany) with 100× objective. Autofluorescing cells at least 0.5 μm in diameter were included in counts and autofluorescing cells smaller than this size were rarely observed. *Synechococcus* cells are typically larger than *Prochlorococcus* (0.9 μm in diameter on average compared to 0.6 μm; Morel et al., 1993) and are known to be relatively more abundant than *Prochlorococcus* in coastal temperate waters (Zwirglmaier et al., 2008). For this reason, cell counts are considered to be a good representation of *Synechococcus* abundance, but we cannot be certain that *Prochlorococcus* did not also contribute to cell counts. Phycoerythrin (PE)-rich cyanobacteria cells autofluoresced yellow to orange, while phycocyanin-rich (PC) cells autofluoresced in red under the above settings.

### Cyanophage Abundance

The abundance of cyanophages capable of infecting *Synechococcus* sp. WH7803 was estimated using a most probable number (MPN) assay (**Table 1**), modified from Marston and Sallee (2003). Briefly, a subsample (60 ml) of each replicate seawater sample was centrifuged to remove large particles and bacteria, and the supernatant was serially diluted with sterile natural seawater medium (SN medium; Waterbury et al., 1986). The dilutions were incubated with exponentially growing *Synechococcus* sp. WH7803 in 48-well microtiter plates at 25°C on a 14:10 h day:night cycle at 19 μE m^-2^ s^-1^ light intensity. After 2 weeks, the plates were visually monitored for lysis (wells with less visible pigmentation than control host-only wells), and the total number of lysed wells was recorded. Estimates of the concentration of infectious cyanophages were determined using the MPN Calculator Build 6 freeware (Curiale, 2004).

### Cyanophage Isolation

After MPN plates had incubated for 3 weeks, the liquid in lysed wells (lysate) was collected. To minimize the chance that wells were inoculated with more than one phage, we collected lysates from dilutions where no more than 50% of the total number of wells had lysed. The lysates were stored in cryogenic vials in the dark at 4°C for up to 12 months prior to purification by plaque assay following Marston and Sallee (2003). To purify, each cyanophage lysate was serially diluted and combined with concentrated *Synechococcus* sp. WH7803 culture and warm (37°C) sterile SN media containing 0.3% washed agar. This mixture was poured over a 0.6% agar SN plate and incubated at 25°C and a 14:10 h day:night cycle. Plaques were never observed on control plates containing host cells only (no viral lysate). Upon the appearance of plaques (typically 4–7 days), a single plaque was picked from each plate and regrown on a WH7803 liquid culture in microtiter plates. Each plaque-purified lysate was then treated with chloroform (final volume 10%) and centrifuged (2000 ×*g* for 10 min) to remove remaining host cells and debris. The supernatant containing a purified cyanophage was transferred to a cryogenic vial for storage in the dark at 4°C. While we aimed to collect approximately 50 isolates per site and sampling date, this was often not possible due to low MPN titers and/or variation in the ability of phages to produce visible plaques on agar plates. A total of 406 isolates was obtained (**Table 1**).

### PCR Amplification and Sequencing

Each plaque purified isolate was used directly as template DNA in PCR reactions. As the majority of culturable cyanophages found in coastal seawater belong to the *Myoviridae* family, we first attempted to amplify the myovirus-specific *g20* gene from each isolate using the primers CPS1.1F (5′-GTA GWA TWT TYT AYA TTG AYG TWG G-3′) and CPS8.1R (5′-ART AYT TDC CDA YRW AWG GWT C-3′; Sullivan et al., 2008). Each *g20* PCR reaction (30 μl) contained 1.5 mM of MgCl_2_, 0.2 μM of each primer, 250 μM of dNTPs, 1× PCR buffer, 1 U of 5-Prime HotMaster Taq polymerase (5-Prime, Gaithersburg, MD, USA), and 1.5 μl of plaque purified cyanophage isolate as template. The reaction parameters were: a denaturing step at 94°C for 3 min and then 34 cycles of 94°C for 45 s, annealing at 48.2°C for 45 s and extension at 72°C for 1 min, followed by a final extension step at 72°C for 4 min.

We attempted to amplify the *g20* gene at least twice (two separate PCR reactions). The first attempt was on the original plaque-purified lysate. If that did not produce a visible PCR product, then the same lysate sample was propagated again on the original WH7803 host strain in order to produce freshly grown lysate. In the event that an isolate did not amplify positively for myoviral *g20* in either of these reactions, we then attempted to amplify the isolate for podovirus-specific DNA polymerase (Podo-*DNApol*) using the forward primer CP-DNAP-349F (5′-CCA AAY CTY GCM CAR GT-3′) and the reverse primer CP-DNAP-533R consisting of equal molar amounts of two primers 533Ra (5′-CTC GTC RTG SAC RAA SGC-3′) and 533Rb (5′- CTC GTC RTG DAT RAA SGC-3′; Chen et al., 2009). Each Podo*-DNApol* reaction (30 μl) reaction contained 2.5 mM MgCl_2_, 100 μM dNTPs, 0.2 μM of each primer, 1× PCR buffer, 1 U of Platinum *Taq* DNA polymerase (Invitrogen, Carlsbad, CA, USA), and 1.5 μl of plaque purified cyanophage isolate as template. A touchdown PCR reaction was performed, consisting of the following parameters: 95°C for 3 min, followed by four cycles of 95°C for 30 s, 49°C for 30 s with a decrease 0.5°C at each step, and 72°C for 1 min; and then 29 cycles of 95°C for 30 s, 47°C for 30 s, and 72°C for 1 min; and a final extension step at 72°C for 10 min. Four isolates did not amplify positively for either gene with the chosen primers and were henceforth removed from further analysis.

Positive *g20* (~540 bp) and Podo*-DNApol* (~550 bp) PCR products were sent to Beckman Coulter Genomics (Beverly, MA, USA) for Sanger sequencing using the forward primer for each primer pair. Eleven myovirus isolates did not yield quality *g20* sequences, even when they produced a visible PCR product. In most of these cases, the PCR product was noticeably weak indicating low phage titer. These 11 isolates were confirmed to be negative for podovirus amplification via the Podo*-DNApol* PCR reaction above, and were therefore assumed to be myoviruses. All podovirus isolates yielded high quality Podo*-DNApol* sequences. Note that the number of total isolates and, therefore, sequences obtained varied both by site and by collection date (**Table 1**).

### Isolate Sequence Analysis

For isolates, nucleotide sequences were edited and aligned for each gene separately, taking into account the amino acid translation using MUSCLE in Geneious 4.6.2 (Auckland, New Zealand). After trimming and editing, the resulting gene fragments were 507 and 492 bp in length for *g20* and Podo*-DNApol*, respectively. To characterize the taxonomic identity of each isolate, we grouped nucleotide sequences into OTUs, at a 99% sequence similarity threshold for *g20* (Supplementary Table S2) and 98% sequence similarity threshold for Podo-*DNApol*. These cutoffs were chosen to be consistent with other work (e.g., Marston and Amrich, 2009; Huang et al., 2010; Clasen et al., 2013), and OTU designations for the podoviruses did not change if a 99% cutoff was used instead. To create OTUs, the nearest-neighbor cluster algorithm in MOTHUR v.1.27 (Schloss et al., 2009) was performed on DNA distance matrices constructed in PHYLIP v.3.68 (Felsentein, 1993) using the F84 distance model. One representative sequence for each OTU was selected using the get.oturep function in MOTHUR and used to build neighbor-joining phylogenetic trees in Mega5 for Mac (Tamura et al., 2007) with bootstrap support calculated from 10,000 replications. OTU representatives were also used for GenBank Blast searches using blastn and blastx batch search functions. Rarefaction curves were created by using Coleman richness estimates from EstimateS (Colwell, 2009). Cyanomyovirus OTUs were named according to conventions, as in Marston and Sallee (2003) and Clasen et al. (2013). Representative sequences from each OTU have been deposited in GenBank under accession numbers JX218109–JX219124 and KU867817–KU867830.

### Characterization of Host Range Phenotypes

To characterize host range phenotypes (i.e., the range of *Synechococcus* host strains that a cyanophage isolate is capable of lysing), we tested the ability of a subset of isolates (*n* = 246) to infect each of 10 different *Synechococcus* host strains (“infectivity assays”). Isolates chosen for the phenotype assays were selected at random from each environmental sample and each OTU having an abundance >3, such that all samples, sites, and major OTUs were represented in the phenotype assays. The number of isolates from each OTU tested for host range was roughly proportional to the relative abundance of those OTUs in the taxonomic composition analysis. Note that host range data for 33 isolates was ultimately discarded from further analyses because the isolate did not pass the control lysis or PCR confirmation tests (described below).

The host strains used in the assays included four wild-type strains: WH8012, WH8018, WH8101, and CC9311; and six strains derived from these wild-type strains, but selected for resistance against previously isolated cyanomyoviruses (Stoddard et al., 2007): WH7803R8R21, WH8018R19, WH8018R34, WH8018R3R2R6, WH8101R3, and WH8101R3R32. These resistant strains have been demonstrated to vary considerably in their susceptibility to cyanomyovirus isolates from Narragansett Bay, RI (Lennon et al., 2007; Stoddard et al., 2007). Therefore, the entire suite of hosts was intended to represent a diversity of host susceptibility phenotypes. All strains contained PE (i.e., “PE-rich”) except WH8101 and its resistant variants, which do not contain PE (i.e., are “PC-rich”). Further information about the WH wild-type and resistant strains can be found in Lennon et al. (2007), while that of CC9311 can be found in Toledo and Palenik (1997). The host strain used for initial isolation (wild-type WH7803) was also included in infectivity assays as a positive control. All cultures were maintained in the same conditions as the strain used for isolation (in SN media at 25°C on a 14:10 h day:night cycle at 19 μE m^-2^ s^-1^ light intensity) except CC9311, which was maintained in SN at 25°C on a 14:10 h day:night cycle at 75 μE m^-2^ s^-1^.

Assays were conducted in 48-well microtiter plates by adding 10 μl of plaque purified cyanophage isolate to each of two duplicate wells containing 200 μl of exponentially growing *Synechococcus* strain. Each plate contained 21 individual phage isolates in duplicate wells along with a row (six wells) of negative controls consisting of host strain only. Plates were allowed to incubate at room temperature under low light conditions for at least an hour to allow for attachment of phages to host cells before 500 μl of SN media was added to each well. Plates were incubated for a total of 4 weeks at 25°C on a 14:10 h day:night cycle at 19 μE m^-2^ s^-1^ light intensity, except for CC9311 plates which were incubated for a total of 3 weeks at 25°C on a 14:10 h day:night cycle at 75 μE m^-2^ s^-1^. After every 7 days (once per week of incubation), or 4 days for CC9311, plates were scored for lysis by visual inspection, and wells that were completely clear (i.e., lacking pigment) compared to pigmented host-only control wells were considered lysed. A virus was considered capable of infecting a host strain and given a score of “1” for that host strain if both of the duplicate test wells were lysed at any point during the incubation period. A virus was considered unable to infect a host strain and given a score of “0” if neither or only one of the duplicate test wells was lysed after 4 or 3 weeks (CC9311) of incubation. This resulted in a binary phenotypic profile for each cyanophage isolate (Supplementary Table S3). While we maintained plates for 3–4 weeks total, no new lysis was observed after the 3rd week for all WH strains or after 12 days for CC9311.

Seventeen of the cyanophage isolates tested did not lyse the positive control strain (WH7803), and thus were removed from further phenotypic analysis. The rest of the assayed phages did lyse the positive control strain (*n* = 229), and at the end of the incubation period, lysates from these control wells were collected and stored in cryogenic vials at 4°C. Presence of phages in these control lysates was confirmed via amplification of *g20* or Podo-*DNApol* as described above. In cases where the expected PCR product could not be obtained from positive control lysates (*n* = 16), the isolate was removed from further phenotypic analysis. This resulted in host range phenotype profiles for 213 isolates total: 183 myoviruses and 30 podoviruses. A random subset of six phage isolates were chosen to repeat the assays, and in each case a phenotypic profile identical to that observed in the initial assay was obtained. Finally, although isolates were purified via plaque purification, we could not be certain that they were pure (i.e., consisting of a mixture with multiple genotypes or with an unknown phage that did not amplify with our primers), as has been noted in other cyanophage studies (e.g., Dekel-Bird et al., 2014). Therefore, we restrict our analysis to comparing overall host range differences between sites and do not examine the phylogenetic relatedness of host range patterns among the isolates.

### Statistical Analyses

To test for differences in total cyanobacteria abundances and the abundances of WH7803-infecting cyanophages among locations (i.e., CA, WA, and RI), a nested analysis of variance (ANOVA) was performed in JMP (version 5.1.2, SAS Institute Inc., Cary, NC, USA) on log-transformed abundance estimates, with site nested within location. Regression analysis was also carried out in JMP on log-transformed data to test for a correlation between the two abundance estimates across all samples.

To compare the composition of cyanophage communities among locations, we conducted non-parametric permutational analyses in PRIMER-E v6 (Plymouth Routines In Multivariate Ecological Research) analysis package (Ivybridge, UK). These analyses are less sensitive to many of the assumptions of parametric methods, including variation in sample size (Clarke, 1993). For tests among locations, sites were used as replicates. For tests among sites within CA, sampling dates were used as replicates. We used multi-dimensional scaling (MDS) ordination to visualize patterns among locations and ANOSIM to test whether composition varies among groups (e.g., between the three locations). ANOSIM tests that the within-group distances are greater than the average of the between-group differences, or “the degree to which there is greater clumping (smaller distances) among samples within the same group compared to that observed among samples in different groups” (Anderson and Walsh, 2013). ANOSIM generates an *R*-statistic ranging from 0 to 1 with greater *R*-values corresponding to greater dissimilarities among groups. We considered *R*-values below 0.3 as weakly or marginally dissimilar even if *p* < 0.05 (Clarke and Warwick, 2001).

Prior to MDS and ANOSIM, similarity matrices were generated as follows. For taxonomic composition of cyanomyovirus isolates, a Bray–Curtis similarity matrix was calculated from OTU relative abundance data. Samples having fewer than five sequences were removed first (PC and the September 2nd samples from both TA and TB) and the OTU-by-sample data were square root transformed. For host range phenotypes, the pairwise crosses between all tested cyanomyovirus isolates and the 10 host strains resulted in a binary phenotype matrix, which was converted into a similarity matrix using the Czekanowski metric (Legendre and Legendre, 1998):

$$S_{ik}\,=\;\frac{1}{p}\,\;\overset{p}{\underset{j=1}{\Sigma }}\left|{\;y}_{ij}-y_{kj\,}\,\;\right|$$

Here, s_ik_ is the similarity in host range profile for phage isolates i and k, j is the first of p total phage isolates, and y is the phenotype for a given isolate. This matrix was used to test for differences in host range phenotypes of myoviruses among sites and locations. A second similarity matrix was generated for the phenotype matrix of all assayed phage isolates to test for phenotypic differences among phage families (*Myoviridae* vs. *Podoviridae*). Note that statistical comparisons among the cyanopodoviruses were not possible due to low sample size and site imbalance that arose by chance through the isolation approach, and thus we report the results for cyanopodoviruses as qualitative descriptions.

### Direct Sequencing of Cyanophage *g43* Diversity

Because the cultivation-dependent approach can target only a subset of the cyanophage diversity present in a sample, we also examined cyanophage community composition by direct pyrosequencing of the myovirus *g43* DNA polymerase gene, using primers targeting cyanophages of the *Myoviridae* family (Marston et al., 2013). Note that this is not the same as either of the genes and primer sets as used above. Sampling and replication was the same as above for the isolation-based approach, except that instead of three separate sites in RI, we used samples collected over thee time points (between August 1 and September 5 2010) at a single site in RI (the RWU site, **Table 1**). These served as replicates for RI. For each of these coastal water samples, 50–200 ml of prefiltered [GF/F filter (Whatman) followed by 0.22 um Sterivex (Millipore)) sample was filtered onto either a 0.02 μm pore size Anodisc filter (Whatman), or a 0.03 or 0.05 μm pore size polycarbonate filter membrane (Sterlitech). Filters were stored at -80°C until DNA was extracted using the Power Water DNA kit (Mo Bio Laboratories). The g43 gene was amplified using concatemers containing the 454 A adapter with a 12 bp tag followed by the g43-For specific primer (Marston et al., 2013). The reverse primer contained the 454 B adapter with a 8 bp tag followed by the g43-Rev specific primer (Marston et al., 2013). The PCR set up used 5 μl of DNA (0.03–5 ng/μl), 1.5 mM MgCl_2_, 50 mM of KCl, 0.25 mg/ml Bovine Serum Albumin (BSA), 1 μM of each primer and 1 U of Taq Polymerase (HotMaster, 5 Prime) and water to a final volume of 25 μl. The PCR program included: 3 min of denaturing at 94°C; 30 cycles of 45 s at 94°C, 1 min at 51°C, 1 min at 72°C; and a final extension of 4 min at 72°C. PCR products were purified by gel extraction using AMPure Beads (Beckman Coulter). Purified PCR products were pyrosequenced at the Duke University IGSP Sequencing Facility (Durham, NC, USA). Sequences were denoised and binned into OTUs using MacQIIME ver 1.9.0. After denoising and removal of singleton OTUs, the dataset consisted of 122,300 reads total in 1373 OTUs over 20 samples. A median Bray–Curtis dissimilarity matrix was created from 100 OTU tables rarified to the smallest sample size (605 sequences). MDS and ANOSIM were performed as described above. Sequences have been deposited in NCBI SRA under accession number PRJNA288142.

## Results

### Cyanophage and Cyanobacteria Abundances

The abundance of cyanophages infectious to WH7803 and the abundance of cyanobacteria cells (**Table 1**) were significantly different among the three locations (nested ANOVA, location: *F*_1_ = 115.1, *p* < 0.0001; and *F*_1_ = 66.8, *p* < 0.0001, respectively). The abundance of WH7803-infecting cyanophages ranged from less than 10 to 6.0 × 10^3^ infectious units ml^-1^, while cyanobacteria abundance ranged from 1.8 × 10^3^ to 7.9 × 10^4^ cells ml^-1^. Abundances of both cyanophages and cyanobacteria were highest in RI (Tukey–Kramer honest significant difference: *Q* = 2.42, *p* = 0.05 and *Q* = 2.43, *p* = 0.05, respectively). While cyanophage abundance was generally lowest for the CA sites at both sampling time points, cyanobacteria abundance at these sites tended to be more intermediate. Instead, the lowest cyanobacteria estimates were observed for the WA sites. Note that abundance of cyanophages infectious to WH7803 is not necessarily indicative of the abundance of *all* cyanophages. Accordingly, there was no significant relationship between the abundance of cyanophages infectious to WH7803 and the total abundance of cyanobacteria across all sites (*R*^2^ = 0.018). PE-rich cells were observed in all samples, but only RI sites contained a detectable mixture of both PE-rich and PC-rich cells. The proportion of PC-rich cells at these sites out of the total cyanobacteria counts (see Materials and Methods) was 26.6% at NT, 60.3% at CP, and 70.8% at RWU.

### Description of Cyanophage Isolates

A total of 406 cyanophage isolates capable of infecting WH7803 was obtained. Using cyanomyovirus-specific *g20* gene primers and cyanopodovirus-specific DNA polymerase (DNApol) gene primers, 358 of these isolates were determined to be myoviruses, 44 were determined to be podoviruses, and another four did not amplify positively for either gene with the chosen primers. While we isolated cyanomyoviruses at all sites in all three locations, we only found cyanopodoviruses at the RI and WA locations. In fact, no podoviruses capable of infecting WH7803 have been isolated at any of the California-Orange County sites over 5 years of monthly sampling or over 2 years of periodic sampling at the California-Tijuana River sites (Clasen et al., 2013; and unpublished data). RI had a greater relative proportion of cyanopodoviruses (32 out of 77 total isolates; 41.5%) compared to WA (12 out of 51 total isolates; 23.5%; **Table 1**).

### Diversity of Cyanomyoviruses

**FIGURE 1:**
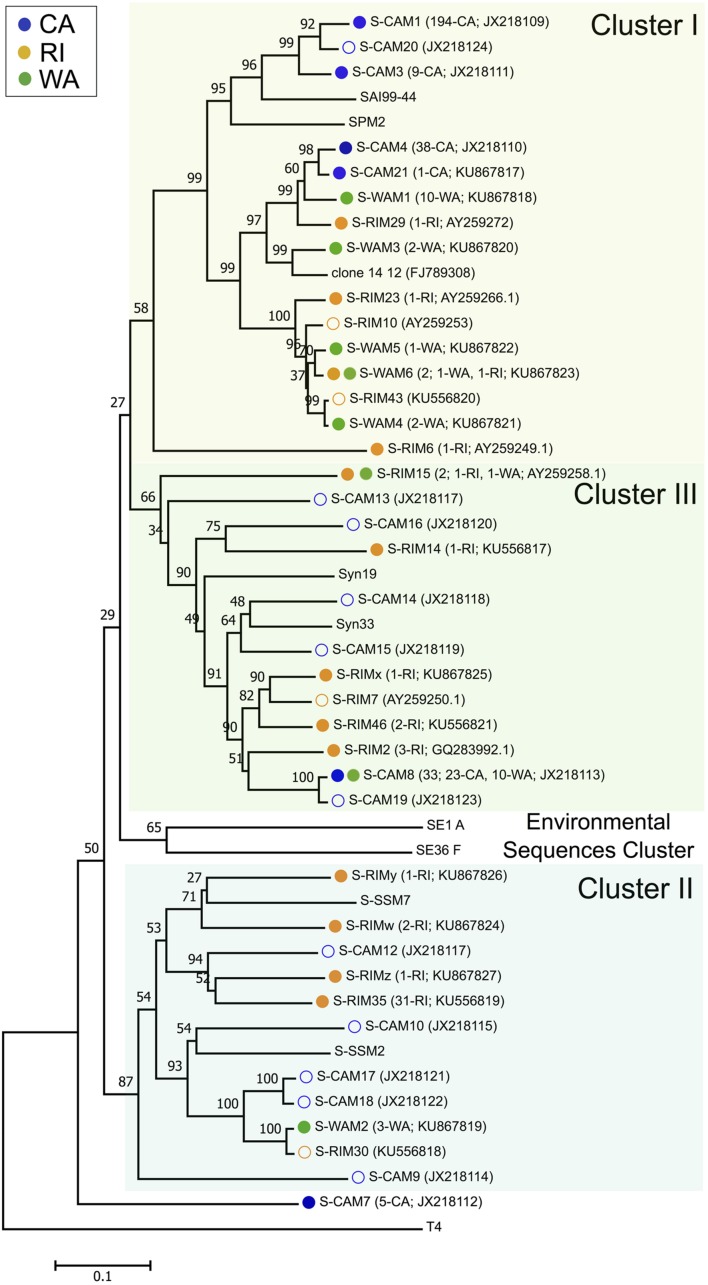
**Neighbor-joining nucleotide tree depicting phylogenetic relationships of isolated cyanomyovirus OTUs based on a 99% sequence similarity cutoff of the *g20* gene.** OTUs containing cyanomyovirus isolates collected in this study are denoted by solid symbols, while open symbols indicate OTUs previously observed at the CA or RI sites. Different colored circles indicate the location of origin (blue, CA; green, WA; and gold, RI) and OTUs consisting of isolates from multiple locations are indicated with two circles. All other taxa are reference sequences. The total number of isolates contained in each OTU is given in parentheses, followed by the location or the number of isolates from each location contained in that OTU, and the GenBank accession number. Major phylogenetic clusters are labeled according to Sullivan et al. (2008).

Three hundred and forty-seven isolated cyanomyoviruses produced quality *g20* sequences, which grouped into 24 total OTUs at a 99% sequence similarity threshold (**Figure 1**). RI was the most OTU-rich, with 47 total isolates belonging to 13 different OTUs. The regions on the Pacific coast were less taxonomically diverse, with the 30 WA myovirus isolates falling into eight OTUs and the 270 CA isolates falling into just six OTUs. Rarefaction curves confirmed that CA cyanomyoviruses are the least OTU-rich (Supplementary Figure S1). RI cyanophage isolates were also more phylogenetically diverse than the WA and CA cyanophages. For example, the RI OTUs detected in this study were widely distributed throughout all three of the major phylogenetic clusters (clusters I–III), while most of the CA and WA OTUs detected in this study belonged to a single cluster, cluster I (as defined in Zhong et al., 2002; Sullivan et al., 2008; **Figure 1**).

### Taxonomic Composition of Cyanomyoviruses

**FIGURE 2:**
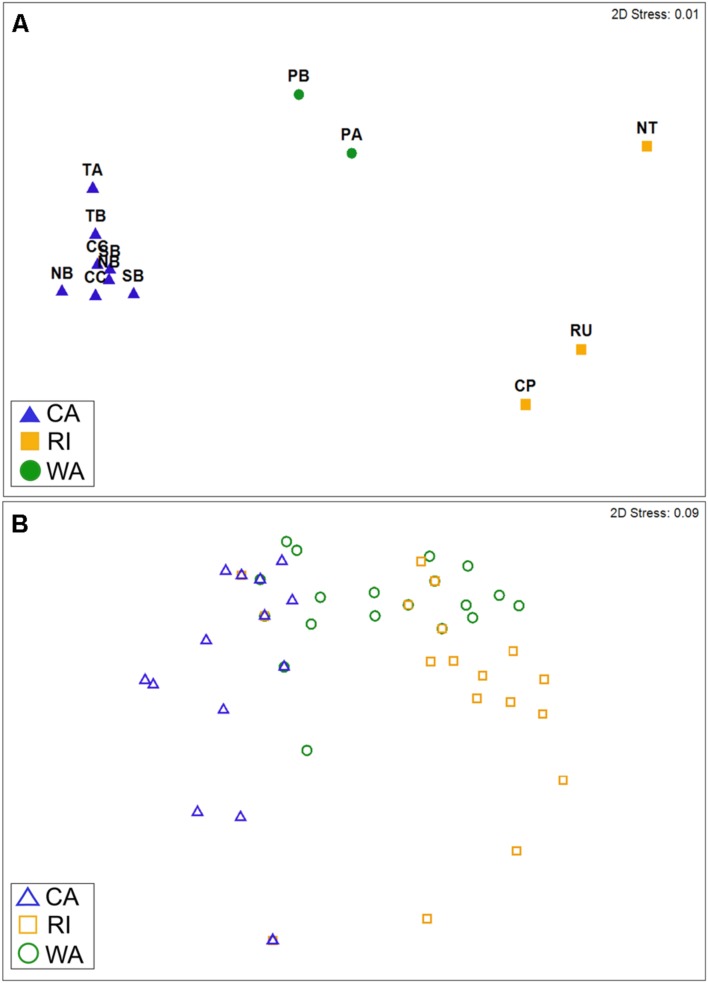
**Non-metric multi-dimensional scaling plots depicting similarity in cyanomyovirus isolates by geographic location based on **(A)** taxonomic composition (Bray–Curtis similarity of OTU composition) or **(B)** host range phenotypes. (A)** Points represent the OTU composition of isolated cyanomyoviruses in each site. For CA, multiple points for each site represent different sampling dates. **(B)** Points represent the host range of individual isolates (*n* = 213) and are shown as open symbols to clarify overlaps (many isolates had identical host range profiles and therefore are completely overlapping in the plot). In both panels, points that are farther apart are more dissimilar from each other in terms of their OTU composition **(A)** or host range phenotype **(B)**.

The taxonomic (OTU) composition of the cyanomyovirus isolates differed significantly by location (ANOSIM: *R* = 0.97, *p* < 0.001; **Figure 2A**). The majority of OTUs were restricted to just one location (i.e., consisted of isolates from one of the three locations). None of the OTUs were shared across all three locations, and only three OTUs were shared by two locations (S-CAM8, S-WAM6, S-RIM15). There were no OTUs shared between CA and RI, but WA had one OTU in common with CA (S-CAM8) and two low-abundance OTUs in common with RI (S-WAM6 and S-RIM15). This small degree of taxonomic overlap between WA and CA and WA and RI likely accounts for the positioning of the WA samples between CA and RI in **Figure 2A**. A similar spatial pattern was also observed by direct pyrosequencing of the whole non-cultured cyanomyovirus community targeting the *g43* gene (ANOSIM by location: *R* = 0.97, *p* < 0.001; Supplementary Figure S2).

### Diversity and Composition of Cyanopodoviruses

**FIGURE 3:**
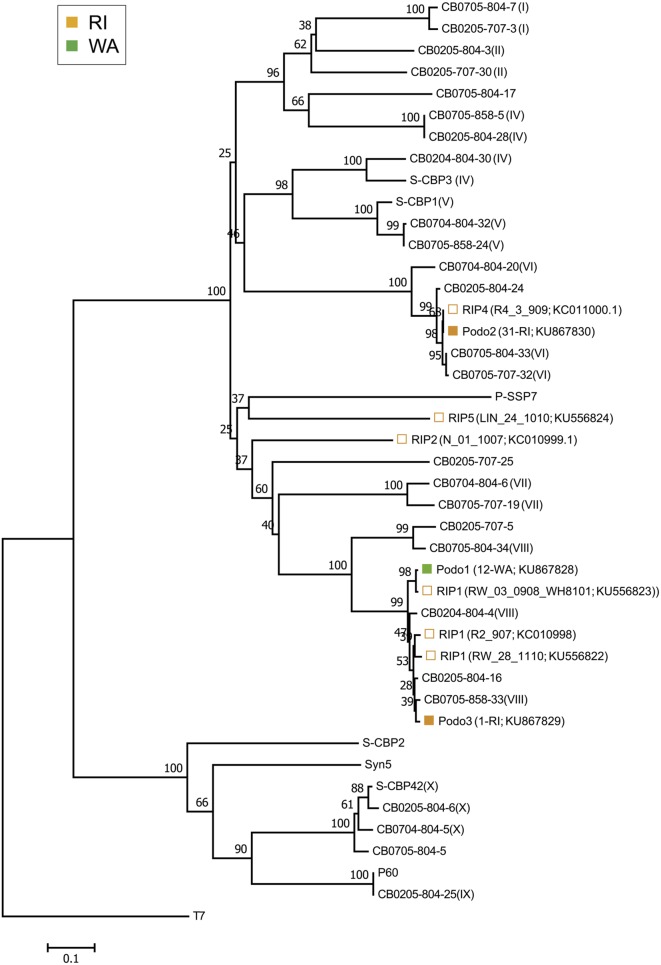
**Neighbor-joining nucleotide tree depicting phylogenetic relationships of isolated cyanopodovirus OTUs based on a 98% sequence similarity cutoff of the DNA polymerase gene.** OTUs-containing cyanopodovirus isolates collected in this study are denoted by solid symbols. Open symbols indicate OTUs previously observed at the RI sites; these are followed by isolate number and GenBank accession number in parentheses. All other taxa represent reference sequences, some of which indicate their phylogenetic subcluster as defined by Huang et al. (2010) as Roman numerals in parentheses. For the podovirus OTUs found in this study, the total number of isolates followed by the source location and GenBank accession number are shown in parentheses. Note that at 98% nucleotide similarity, the Podo1 and the Podo2 OTUs are identical to RIP1_V08_3016_908 and RIP4, respectively, but were included separately for emphasis.

The isolated cyanopodoviruses were less diverse than the isolated cyanomyoviruses, forming just three OTUs at the 98% sequence similarity cutoff and spanning two phylogenetic clusters (**Figure 3**). The Podo1 OTU consisted of all 12 podovirus isolates from WA, but had a *DNApol* sequence nearly identical (99.6%) to that of a cyanopodovirus isolate previously observed in RI (RIP1). The Podo2 OTU consisted of all but one of the podoviruses isolated from RI in this study, and was 100% identical in *DNApol* sequence to that of an isolate previously observed in the same area (RIP4). Podo3 consisted of one isolate from RI, and was more closely related to the WA-specific OTU, Podo1, than to Podo2.

### Host Range Phenotypes

We found that cyanophage isolates also varied in their host range phenotypes as determined by their ability to infect 10 different host strains, even though they all shared the ability to infect the strain used for isolation (WH7803; Supplementary Table S3). Among the myovirus isolates alone, host range phenotypes were significantly different by location (ANOSIM: *R* = 0.62, *p* < 0.001; **Figure 2B**), with all pairwise location comparisons significant (pairwise *post hoc* tests all *p* < 0.001). Further, host range phenotypes differed significantly between sites within CA [ANOSIM: *R* = 0.15, *p* < 0.001; all *post hoc* pairwise tests significant at *p* < 0.05 except Crystal Cove State Park (CC)–Newport Beach Pier (NB)]. Cyanomyoviruses from CA and RI exhibited the most dissimilar host ranges (*post hoc* pairwise comparisons: *R* = 0.81, *p* < 0.001; **Figure 2B**). It is important to note that our analysis of host range phenotypes was restricted to overall site comparisons representing the “community host range” (rather than on an individual isolate level), due to uncertainty in the absolute purity of isolates (see Materials and Methods).

**FIGURE 4:**
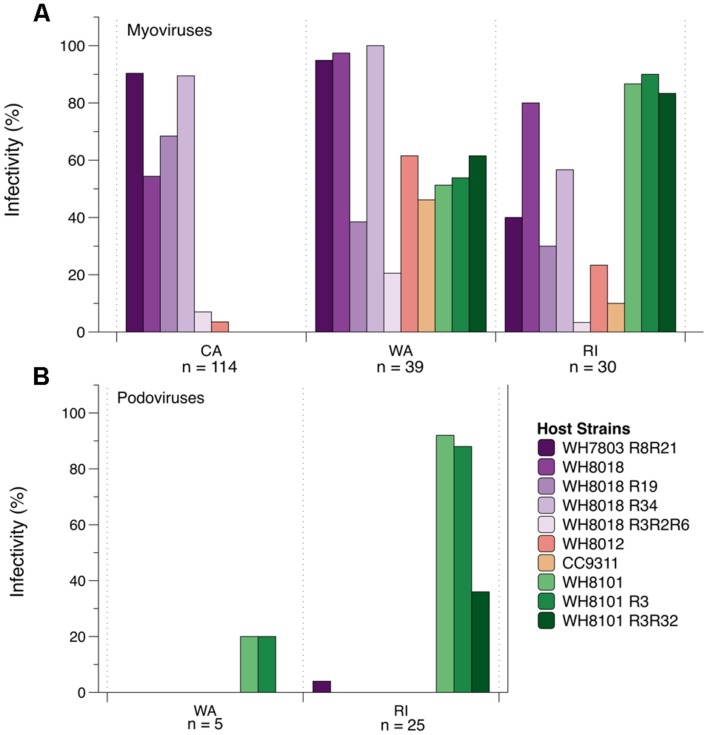
**The percent ability of isolated cyanomyoviruses **(A)** and cyanopodoviruses **(B)** from each geographic location to infect 10 different *Synechococcus* host strains**.

The CA cyanomyovirus community as a whole was capable of infecting fewer hosts as compared to the communities from WA and RI (**Figure 4A**). Only 6 out of 10 host strains were susceptible to myovirus isolates from CA and none of the 114 CA myovirus isolates tested were capable of infecting WH8101 or its resistant variants, compared to 50–60% of the WA isolates and 80–90% of RI isolates. Additionally, very few of the CA isolates could infect WH8012 (4%) compared to 61 and 23% for WA and RI, respectively. Further, none of the 114 CA isolates could infect strain CC9311, a strain originating from the Pacific Ocean off the coast of southern CA (Toledo and Palenik, 1997), compared to 46% of the isolates from WA and 10% from RI that were capable of infecting this strain.

In contrast to the generally narrow host range of the CA isolates, the myoviruses from WA and RI were collectively capable of infecting all 10 of the host strains on which they were tested (**Figure 4A**), yet no single isolate was capable of infecting all 10 host strains (Supplementary Table S3). Nevertheless, host ranges were significantly different between WA and RI (ANOSIM *post hoc* comparisons: *R* = 0.30, *p* < 0.001), as the relative proportion of isolates from each location that could infect certain strains was variable (**Figure 4A**). For example, WA isolates were much more likely to infect WH7803R8R21 and WH8018R34 than RI isolates (95–100% compared to 40–56%; **Figure 4A**), while RI isolates were much more likely to infect WH8101 and its variants (83–90% compared to 51–62%).

The host range phenotypes of myovirus isolates were also significantly dissimilar from that of the podovirus isolates (ANOSIM: *R* = 0.70, *p* < 0.001). Qualitatively, the podoviruses from both WA and RI were capable of infecting fewer of the hosts as compared to the myoviruses (**Figure 4B**). In particular, none were able to infect WH8018 and its resistant variants, WH8012, or CC9311. They could, however, infect WH8101 and its resistant variants, and 1 out of the 30 podoviruses tested could infect the resistant variant of WH7803. RI and WA tended to differ qualitatively in the ability of their podoviruses to infect WH8101 and its variants. Only one out of five WA podoviruses tested could infect WH8101 and WH8101R3, while none could infect 8101R3R32. By contrast, 92, 88, and 36% of the 25 RI podoviruses tested could infect 8101, 8101R3, and 8101R3R32, respectively.

## Discussion

Overall, we found evidence for spatial structure in the composition of coastal marine cyanophage communities isolated from three coastal regions in the U.S.—one on the Atlantic and two on the Pacific coast. Moreover, differentiation at this spatial scale was observed at both a taxonomic level and a phenotypic level. Cyanophages from each location differed significantly in taxonomic composition as well as their host range phenotypes. Regardless of how diversity was defined, however, CA and RI cyanophage communities were the most divergent, while WA communities exhibited some similarities to each of the other two. Even though most taxa were unique to a particular location, a few taxa were shared between WA and CA and between WA and RI. Strikingly, no taxonomic overlap of isolates was observed between CA and RI. The presence of podoviruses infectious to WH7803 in WA and RI, but not in CA, further supports this general spatial pattern.

Contrary to our hypothesis, the taxonomic composition of cyanophage communities exhibited just as much spatial structure as did the composition of host range phenotypes at the spatial scale of our study. However, a pattern consistent with our initial hypothesis might be observed at smaller spatial scales than represented by the sampling locations in this study, because the phage–host interactions presumably responsible for variation in host range phenotypes occur at very local spatial scales. In support of this, studies in non-marine systems indicate that spatially explicit adaptation of phages to local host communities occurs at very small spatial scales (e.g., within several centimeters in soil or within a single tree; Vos et al., 2009; Koskella et al., 2011). Indeed, within CA, we found that host range phenotypes varied marginally at smaller spatial scales (among sites) while the same comparison for taxonomic composition was not significant (ANOSIM by site within CA for host range: *R* = 0.15, *p* < 0.001; for OTU composition: *R* = 0.12, *p* = 0.13). It is also possible that the amount of phenotypic variability we were able to detect was limited by the set of hosts used for our host range assays, especially if they were not representative of naturally co-occurring host diversity. More *Synechococcus* isolates from our sampling regions would be needed to fully test this possibility.

Here we report biogeographic structure in host range phenotypes among a large number of individual cyanophages isolated across a regional scale. A handful of other studies have investigated host range dynamics for marine phages in a biogeographic context (Moebus and Nattkemper, 1981; Wichels et al., 2002; Comeau et al., 2005, 2006; Holmfeldt et al., 2007; Flores et al., 2013; Castillo et al., 2014; Dekel-Bird et al., 2014), often reporting a spatial, temporal, or environmental signature in host infectivity. Our study is unique in that it compares the host range phenotypes and taxonomic composition of a large number of marine cyanophage isolates (*n* = 213 for host range, *n* = 406 for taxonomy) collected over a relatively large spatial scale (approximately 1–4000 km apart). Although host range information is a first step toward understanding the ecological role of phages and their host interactions, few recent studies perform such phenotypic assessments likely because doing so depends on the cultivation and isolation of both phages and hosts. Several new cultivation-independent techniques, such as viral tagging (Deng et al., 2012, 2014) and phageFISH (Dang and Sullivan, 2014) offer promising alternatives for studying phage–host interactions in natural samples (Brum and Sullivan, 2015). Such methods could allow for host preference to be characterized among many more phages and samples than is feasible by isolation-based methods. Despite the limitations of using an isolation-based approach, however, the taxonomic composition of our isolates shows a similar biogeographic pattern to that of environmental sequences (Supplementary Figure S2), suggesting that our isolates represent a sufficient subset of diversity for detecting more general distribution patterns in cyanophages.

Phage diversity, abundance, and distribution may be at least partially influenced by that of their host communities (Thingstad and Lignell, 1997; Brum et al., 2015; Huang et al., 2015). In this scenario, bacterial host taxa or genotypes vary in their competitive ability under spatially variable environmental factors, which results in spatially differentiated host communities. As long as phages vary in their host ranges or host specificities, phage communities then effectively “track” the presence and relative abundance of susceptible host strains (Borsheim, 1993). In other words, local host composition selects for cyanophage composition. In fact, the composition of marine *Synechococcus* communities does vary spatially (Fuller et al., 2006; Zwirglmaier et al., 2008; Paerl et al., 2011; Mazard et al., 2012; Sohm et al., 2016), indicating that indirect environmental selection via the host community could certainly play a role in phage distribution. Preliminary analysis of host (*Synechococcus*) composition in our samples indicates that the cyanobacterial communities do indeed differ among the locations, with CA and RI *Synechococcus* communities appearing to be the most dissimilar from each other (Supplementary Figure S3). The finding that host infectivity varies among these locations further lends support to the idea that spatial variation in host communities also influences phage community composition.

The culture-independent analysis of *g43* diversity from the same samples demonstrates that our isolation approach captures similar spatial differentiation of cyanophage composition at our sites (Supplementary Figure S2). Moreover, the five most common isolate OTUs observed at the CA sites in this study were also the most common at these sites in monthly samples collected over a complete annual cycle (Clasen et al., 2013). Thus, the 1-month sampling window of this study successfully sampled the dominant culturable cyanophage taxa present at the CA sites. Still, the cyanophage isolates most certainly represent a selective view of the total cyanophage diversity present. For instance, it is notable that isolate diversity was higher in RI than the Pacific locations. We hypothesize that this pattern is related to the overall higher abundances of cyanobacteria observed in RI during the sampling period (**Table 1**); positive relationships between cyanophage diversity and host abundance have been observed previously (Wang and Chen, 2004; Mühling et al., 2005). However, the choice of isolate host (WH7803) may also be partly responsible for the higher diversity detected; there may be more cyanophages present in RI that can infect this particular host strain.

Spatial structure among the cyanopodoviruses was not as striking as it was for the cyanomyoviruses. While the taxonomic and phenotypic composition of cyanopodoviruses in RI and WA tended to appear qualitatively different from each other, this trend was the result of just two prominent OTUs. Additionally, a third OTU consisting of just one isolate from RI was nearly taxonomically identical to the podovirus OTU found in WA. This pattern suggests that marine cyanopodoviruses are widespread in their distribution and less diverse than cyanomyoviruses, as previously suggested (Huang et al., 2010). In their global survey of uncultured podovirus-specific *DNApol* sequences, Huang et al. (2010) also found that cyanopodoviruses belonging to subcluster VIII were the most abundant types found in both the open ocean and in coastal Chesapeake Bay samples. Many of our cyanopodovirus isolates also grouped into this globally abundant subcluster.

## Conclusion

This study demonstrates that marine phages exhibit biogeographic patterns not only in their taxonomic composition, but also in their host range phenotypes. Spatial structure in host range suggests that phage infection abilities differ in space. Thus, host range differences may contribute to spatial variation in the composition and mortality rates of bacterial communities (Martiny et al., 2014). We speculate that the biogeographic patterns observed here are a combined result of spatial variation in interactions with hosts occurring at local scales and abiotic factors that span larger scales. Our findings lend further support to the growing evidence that phages in natural environments exhibit biogeographic structure driven in part by interactions with their host communities (Morgan et al., 2005; Held and Whitaker, 2009; Vos et al., 2009; Koskella et al., 2011; Marston et al., 2012; Brum et al., 2015), despite high rates of dispersal in ocean currents.

## Author Contributions

The study was conceived and designed by all authors. CH conducted environmental sampling in California and Washington, performed all laboratory and computational analyses, and wrote the paper with input from MM and JM. MM conducted environmental sampling in Rhode Island.

## Conflict of Interest Statement

The authors declare that the research was conducted in the absence of any commercial or financial relationships that could be construed as a potential conflict of interest.
