# Supplementary material for: Biogeographic Variation in Host Range Phenotypes and Taxonomic Composition of Marine Cyanophage Isolates
Source: Front Microbiol. 2016 Jun 24;7:983. doi: 10.3389/fmicb.2016.00983 (PMC4919323; doi:10.3389/fmicb.2016.00983)
Supplement: Supplementary file 1 [file Presentation_1.PDF]

Supplementary Material

Biogeographic variation in host range phenotypes and taxonomic composition of marine cyanophage isolates

China A. Hanson*, Marcia F. Marston, and Jennifer B. H. Martiny

***Correspondence:** Dr. China A. Hanson, School of Biological and Chemical Sciences, Queen Mary University of London, Fogg Building, Mile End Road, London, E1 4NS, UK
c.hanson@qmul.ac.uk

Contents: Figures S1-S3

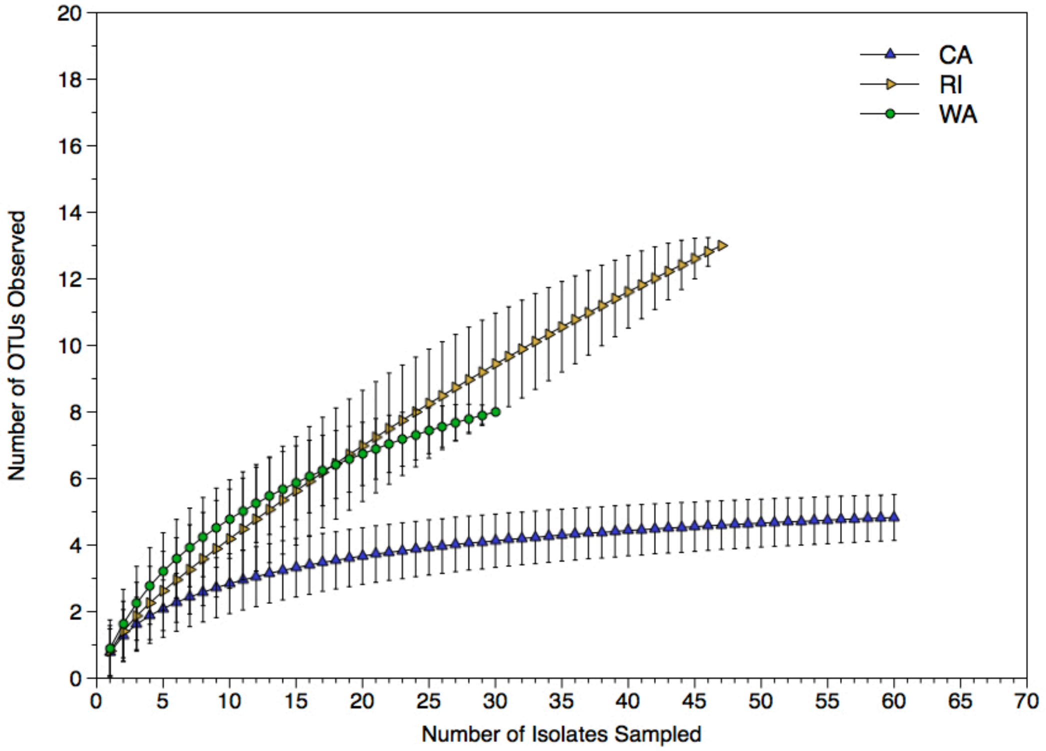

Figure S1. Coleman rarefaction curves for cyanomyovirus operational taxonomic units (OTUs) defined at ≥99% nucleotide sequence similarity of *g20* partial gene sequences. Error bars represent ±SD. For CA, richness estimates from the first 60 sequences only were plotted.

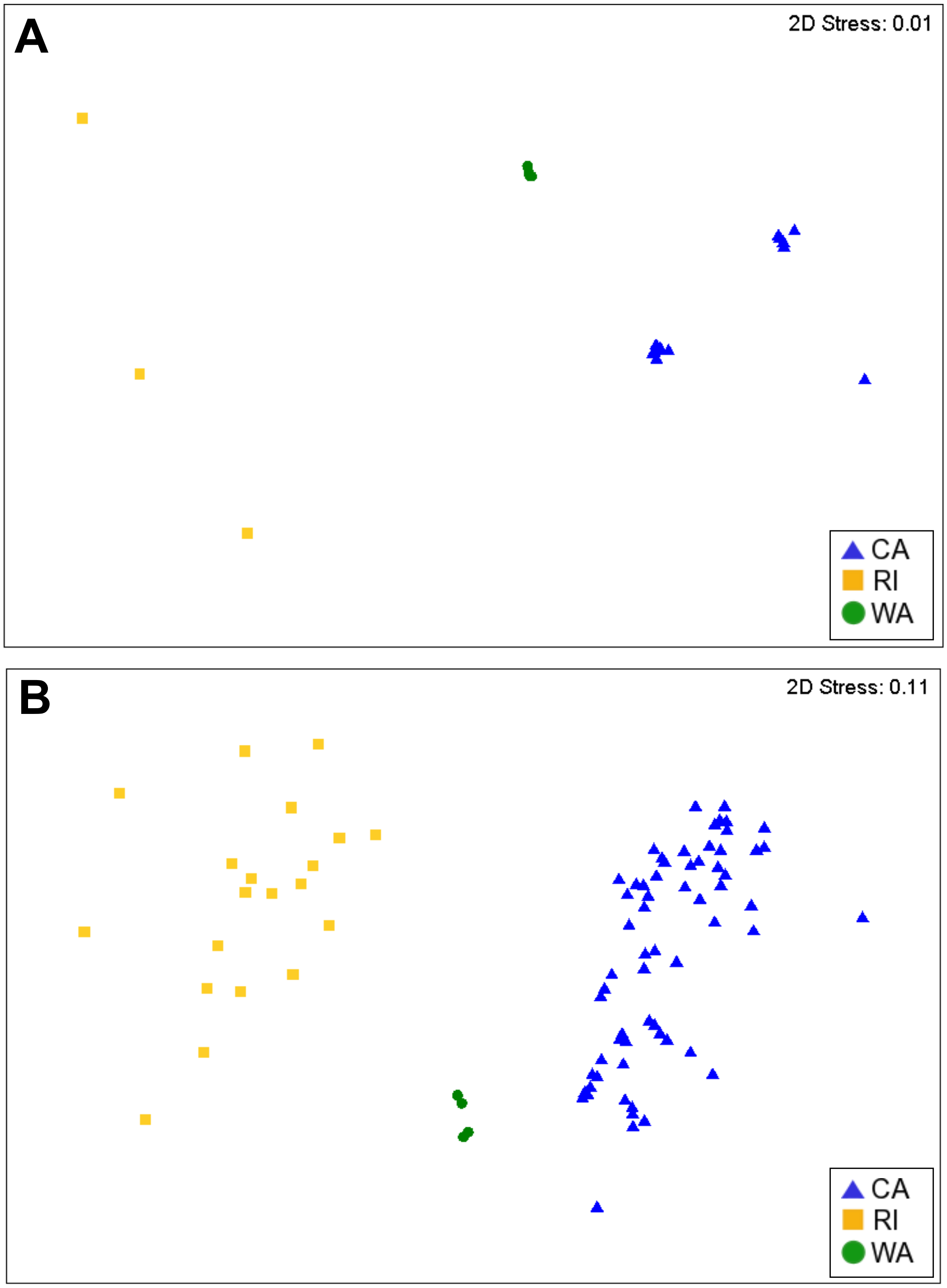

Figure S2. Non-metric multidimensional scaling (MDS) plots for the community composition of whole cyanomyovirus communities based on direct pyrosequencing of the *g43* gene. Each point represents the *g43* OTU composition detected in each sample from each of the three locations. (A) MDS for samples collected in August-September 2010 only. (B) MDS showing that the differences by location are upheld even when a larger set of samples collected monthly over 2 years at CA and RI (January 2010- December 2011) is included.

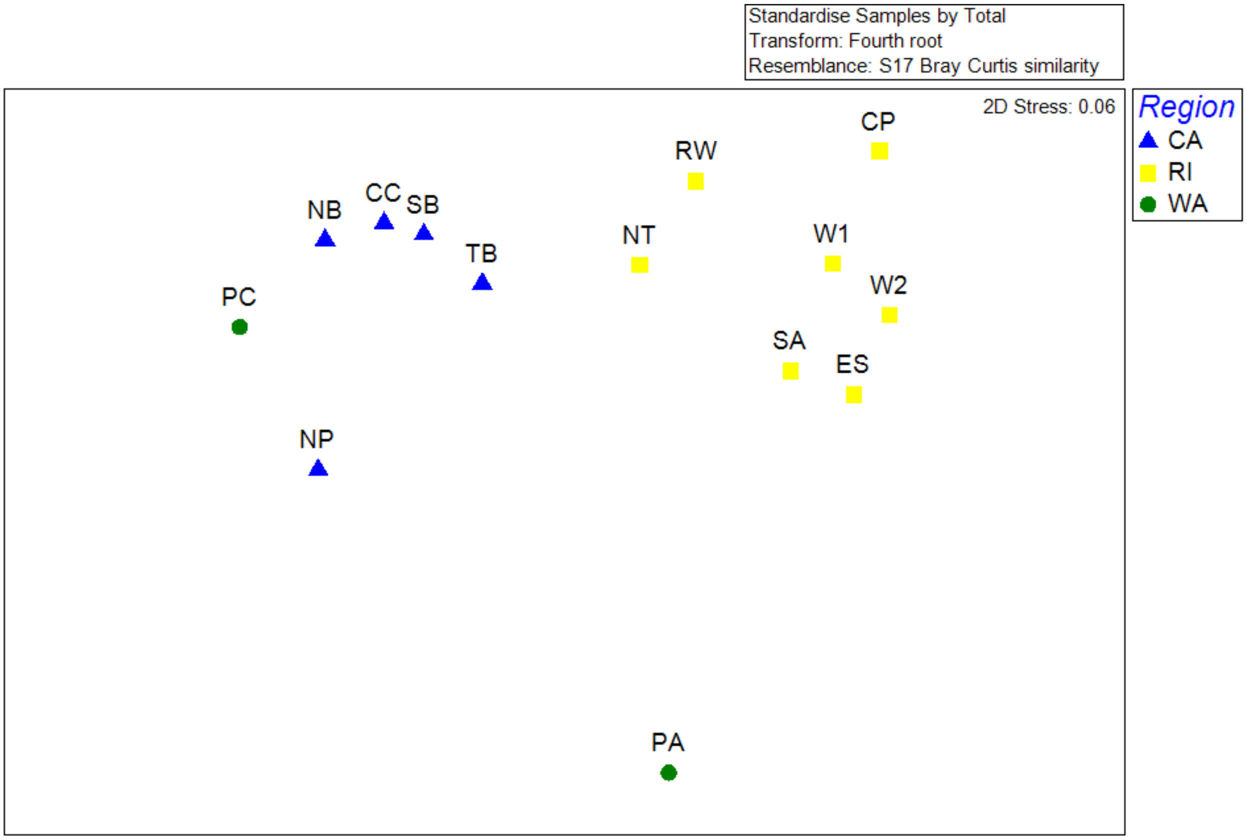

Fig. S3 Non-metric multidimensional scaling (MDS) plot for the community composition of *Synechococcus* communities based on direct pyrosequencing of the *rpoC1* gene. Each point represents the *Synechococcus* OTU composition (99% sequence similarity cutoff of the *rpoC1* gene) detected in each sampling site, color-coded by locations. Additional RI sites not in the main text are: W1 and W2, Westerley 1 and 2; SA Sandwich; ES East Sandwich. Further details about these sites can be found in Marston et al. 2013 (DOI: 10.1111/1462-2920.12062). Prior to MDS, the lowest sample sizes were removed, and a Bray-Curtis similarity matrix was calculated on standardized (by total number of sequences per sample) and fourth root transformed OTU-by-sample data. Sequences are deposited in NCBI SRA under accession number SRP074612.
