# Supplementary material for: Biogeographic Variation in Host Range Phenotypes and Taxonomic Composition of Marine Cyanophage Isolates
Source: Front Microbiol. 2016 Jun 24;7:983. doi: 10.3389/fmicb.2016.00983 (PMC4919323; doi:10.3389/fmicb.2016.00983)
Supplement: Supplementary file 2 [file Data_Sheet_1.DOCX]

**Table S1**. Chemical and physical characteristics measured at each site at the time of sampling.

| **Location** | **Sampling Site** | **Date Sampled** | **Water Temp (°C)** | **Salinity (ppt)** | **pH** | **Phosphate (μM)** | **Nitrate + Nitrite (μM)** | **Nitrate (μM)** | **Ammonia (μM)** | **Silicate (μM)** |
| --- | --- | --- | --- | --- | --- | --- | --- | --- | --- | --- |
| **California** | Crystal Cove State Park (CC) | 8/18/2010 | 15.5 | 36 | 7.3 | 0.35 | 1.92 | 0.14 | 1.40 | 2.43 |
|  |  | 9/15/2010 | 15.2 | 36 | 6.8 | 3.81 | 1.81 | 0.10 | 1.67 | 3.48 |
|  | Newport Beach Pier (NB) | 8/18/2010 | 16.8 | 40 | 7.7 | 0.11 | 1.11 | 0.67 | 3.09 | 0.74 |
|  |  | 9/15/2010 | 15.4 | 38 | 7.2 | 0.37 | 2.57 | 0.21 | 2.08 | 4.04 |
|  | Seal Beach (SB) | 8/18/2010 | 19.2 | 37 | 7.8 | 0.44 | 2.58 | 0.59 | 3.61 | 3.04 |
|  |  | 9/15/2010 | 17.9 | 36 | 8.4 | 0.77 | 3.37 | 0.56 | 3.30 | 5.81 |
|  | Tijuana River, Boca Rio Channel (TA) | 9/2/2010 | 18.4 | 36 | 7.7 | 2.27 | 0.58 | 0.11 | 9.18 | 22.30 |
|  |  | 9/20/2010 | 14.6 | 36 | 7.9 | 1.84 | 9.14 | 0.43 | 14.14 | 17.15 |
|  | Tijuana River, Surf Zone (TB) | 9/2/2010 | 15.0 | 37 | 8 | 0.86 | 2.74 | 0.19 | 7.77 | 9.71 |
|  |  | 9/20/2010 | 14.0 | 37 | 8.1 | 1.20 | 9.39 | 0.38 | 7.19 | 14.10 |
| **Washington** |  |  |  |  |  |  |  |  |  |  |
|  | Washington Park (PA) | 8/20/2010 | 12.5 | 31 | 7.8 | 1.56 | 12.66 | 0.34 | 2.11 | 36.61 |
|  | Samish Island (PB) | 8/20/2010 | 17.4 | 30 | 8.8 | 1.42 | 1.70 | 0.16 | 2.54 | 19.55 |
|  | Padilla Bay (PC) | 8/20/2010 | 24.2 | 28 | 9 | 2.98 | 0.43 | 0.15 | 2.25 | 36.04 |
| **Rhode Island** |  |  |  |  |  |  |  |  |  |  |
|  | Colt State Park (CP) | 9/9/2010 | 22.0 | 30 | 7.9 | 0.65 | 0.21 | 0.21 | 1.35 | 37.34 |
|  | Newport (NT) | 9/9/2010 | 22.0 | 34 | 8.4 | 0.67 | 0.14 | 0.14 | 0.80 | 4.02 |
|  | Roger Williams University (RU) | 9/9/2010 | 22.7 | 32 | 7.9 | 1.05 | 0.30 | 0.30 | 1.90 | 28.99 |
